# Using Natural Language Processing to Examine the Uptake, Content, and Readability of Media Coverage of a Pan-Canadian Drug Safety Research Project: Cross-Sectional Observational Study

**DOI:** 10.2196/13296

**Published:** 2020-01-14

**Authors:** Hossein Mohammadhassanzadeh, Ingrid Sketris, Robyn Traynor, Susan Alexander, Brandace Winquist, Samuel Alan Stewart

**Affiliations:** 1 Dalhousie University Halifax, NS Canada; 2 Nova Scotia Health Authority Halifax, NS Canada; 3 University of Saskatchewan Saskatoon, SK Canada

**Keywords:** natural language processing, mass media, readability, pharmacoepidemiology, knowledge translation

## Abstract

**Background:**

Isotretinoin, for treating cystic acne, increases the risk of miscarriage and fetal abnormalities when taken during pregnancy. The Health Canada–approved product monograph for isotretinoin includes pregnancy prevention guidelines. A recent study by the Canadian Network for Observational Drug Effect Studies (CNODES) on the occurrence of pregnancy and pregnancy outcomes during isotretinoin therapy estimated poor adherence to these guidelines. Media uptake of this study was unknown; awareness of this uptake could help improve drug safety communication.

**Objective:**

The aim of this study was to understand how the media present pharmacoepidemiological research using the CNODES isotretinoin study as a case study.

**Methods:**

Google News was searched (April 25-May 6, 2016), using a predefined set of terms, for mention of the CNODES study. In total, 26 articles and 3 CNODES publications (original article, press release, and podcast) were identified. The article texts were cleaned (eg, advertisements and links removed), and the podcast was transcribed. A dictionary of 1295 unique words was created using natural language processing (NLP) techniques (term frequency-inverse document frequency, Porter stemming, and stop-word filtering) to identify common words and phrases. Similarity between the articles and reference publications was calculated using Euclidian distance; articles were grouped using hierarchical agglomerative clustering. Nine readability scales were applied to measure text readability based on factors such as number of words, difficult words, syllables, sentence counts, and other textual metrics.

**Results:**

The top 5 dictionary words were *pregnancy* (250 appearances), *isotretinoin* (220), *study* (209), *drug* (201), and *women* (185). Three distinct clusters were identified: Clusters 2 (5 articles) and 3 (4 articles) were from health-related websites and media, respectively; Cluster 1 (18 articles) contained largely media sources; 2 articles fell outside these clusters. Use of the term *isotretinoin* versus *Accutane* (a brand name of isotretinoin), discussion of pregnancy complications, and assignment of responsibility for guideline adherence varied between clusters. For example, the term *pregnanc* appeared most often in Clusters 1 (14.6 average times per article) and 2 (11.4) and relatively infrequently in Cluster 3 (1.8). Average readability for all articles was high (eg, Flesch-Kincaid, 13; Gunning Fog, 15; SMOG Index, 10; Coleman Liau Index, 15; Linsear Write Index, 13; and Text Standard, 13). Readability increased from Cluster 2 (Gunning Fog of 16.9) to 3 (12.2). It varied between clusters (average 13th-15th grade) but exceeded the recommended health information reading level (grade 6th to 8th), overall.

**Conclusions:**

Media interpretation of the CNODES study varied, with differences in synonym usage and areas of focus. All articles were written above the recommended health information reading level. Analyzing media using NLP techniques can help determine drug safety communication effectiveness. This project is important for understanding how drug safety studies are taken up and redistributed in the media.

## Introduction

### Web-Based Health Information and News Media

Easy access to health-related information has rapidly transformed the traditional health care delivery paradigm. Patients increasingly use the internet to seek health information and learn more about symptoms, diseases, treatments, self-management, risk mitigation strategies, and shared decision-making with their health care providers [1]. Up to 35% of all adults in the United States (and up to 45% of women and people with higher education) consulted the internet for health or medical information, either for themselves or someone else [2]. In the United Kingdom, 87% of adults read either electronic or traditional newspapers [3]. In 2012, 66.8% of Canadians aged 16 years and older searched the Web for medical or health-related information per Statistics Canada’s *Canadian Internet Use Survey* [4].

News media can have a significant impact on people’s perception and interpretation of scientific research. Journalists and science writers present the results from scientific publications in news articles for the public, health care providers, and policymakers, but also may influence attitudes and health behaviors [5]. Although some believe that the process of journalism is relatively linear with information received from researchers and transmitted by journalists to a poorly informed public, others discuss the cocreation of media with journalists and the public, voluntary health organizations, or professionals in health services delivery, government, and private sector health care companies [3]. News media have guidelines and ethical principles for reporting [6,7], as well as resources to help them interpret the technical material (eg, Evidencenetwork.ca and HealthNewsReviews.org) and review criteria for elements to include in health reporting [5,8]. In addition, organizations such as the Health and Medicine Division of the National Academies of Sciences, Engineering, and Medicine have provided information on communicating the risk, benefit, and uncertainty related to drug therapy [9]. Nevertheless, it has been reported that some media interpretation may be hard to comprehend, fail to provide context, or contain exaggeration, false impression, incorrect numbers, immature data, or not-yet approved methods from ongoing research [3,10-13]. It is, therefore, critical to study how the media cover medical research and investigate the quality of reporting and presentation of scientific findings [14,15].

The use of natural language processing (NLP) techniques and readability assessments can help us better understand how the media are reporting on the medical research we conduct. We used a study conducted by the Canadian Network for Observational Drug Effect Studies (CNODES) evaluating the effectiveness of one aspect of the isotretinoin Pregnancy Prevention Program in Canada [16] as a case study to explore how the media present pharmacoepidemiological research.

### Canadian Network for Observational Drug Effect Studies

CNODES is a network of Canadian pharmacoepidemiologists—distributed across 7 provincial sites and supported by 4 collaborative teams working across all sites—funded by the Canadian Institutes of Health Research (CIHR) to study the risks and benefits of postmarketed drugs [17]. CNODES responds to queries on drug safety and effectiveness from decision makers and other stakeholders (eg, Health Canada and federal, provincial, and territorial pharmacare decision makers) by using meta-analytic methods to combine deidentified administrative health data from across Canada, the United Kingdom, and the United States [18]. The CNODES knowledge translation team leads the network’s activities related to translating and mobilizing research results from specific studies for decision makers, stakeholders, and the public via the media. The results of the CNODES isotretinoin study, described below and published in the *Canadian Medical Association Journal* (CMAJ) in April 2016 [16], were shared via a press release, subsequent media interviews with lead investigators, and a podcast developed by CMAJ to accompany the publication.

### Case Study: Isotretinoin and Pregnancy Prevention Program Adherence

Isotretinoin, a known and potent teratogen, is widely used to treat cystic acne. Fetal exposure may result in a range of severe congenital anomalies and may increase the risk of spontaneous and induced abortion [19,20]. Although the risks of pregnancy during isotretinoin therapy are well recognized, research continues to reveal poor adherence to pregnancy prevention guidelines and programs [21-24]. In Canada, a voluntary pregnancy prevention program was designed to prevent fetal exposure to isotretinoin. It requires informed written consent, 2 pregnancy tests with negative results before starting isotretinoin, and 2 reliable forms of contraception during treatment [25]. The objective of the CNODES study [16] was to evaluate specific aspects of the effectiveness of the Canadian pregnancy prevention program in 4 provinces: British Columbia, Saskatchewan, Manitoba, and Ontario.

In total, 59,271 female patients received 102,308 courses of isotretinoin therapy. Oral contraceptive use during treatment ranged from 24.3% to 32.9%. Overall, there were between 186 and 367 pregnancies during isotretinoin treatment (3.1-6.2 per 1000 isotretinoin users), depending on the method used to define pregnancy. When follow-up was extended to include the full gestational period (up to 42 weeks), there were 1473 pregnancies (24.9/1000 users) using the high specificity definition. Most of these (1331 pregnancies, or 90.4%) were lost spontaneously or terminated by medical intervention. A total of 118 live births were identified and 11 (9.3%) had a diagnosis of congenital malformation. Annual rates of pregnancy during isotretinoin therapy did not change between 1996 and 2011. The CNODES study concluded that adherence to the isotretinoin pregnancy prevention program was poor during the 15-year period [16].

### Objectives of This Study

This study examined media representation and uptake of the CNODES study on the occurrence of pregnancy and pregnancy outcomes during isotretinoin therapy. The specific objectives of this study were to use NLP and other text-analytic methods to: (1) summarize and comprehend the content of the media coverage; (2) identify relationships between the media articles; and (3) analyze the reading levels of the media articles. By obtaining these preliminary objectives, we aimed to explore potential improvements in the way we present future research.

## Methods

### Search Strategy and Media Sources

**Figure 1:**
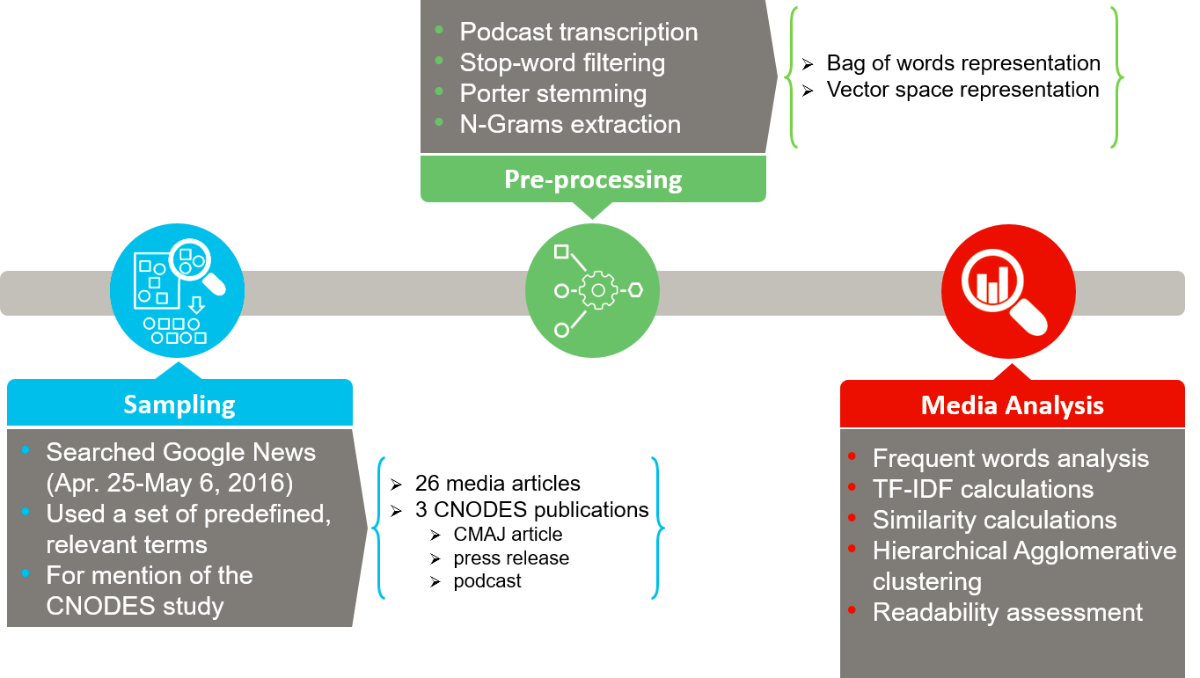
Methodology schematic for our study. CMAJ: *Canadian Medical Association Journal*; CNODES: Canadian Network for Observational Drug Effect Studies; TF-IDF: term frequency-inverse document frequency.

Our overall study methodology is depicted in Figure 1. We conducted a search in Google News from April 25 to May 6, 2016, using a predefined set of relevant search terms, to identify the traditional media sources (eg, local, national, and international news sources) reporting the CNODES isotretinoin study [16], but excluding social media sources. We used the following search strategy: (isotrétinoïne OR Accutane OR Clarus OR Epuris OR isotretinoin OR CNODES OR “Canadian Network for Observational Drug Effect Studies”). We also tracked the media sources captured on the *News* tab on the Altmetric.com page for this article [26], although these sources were all also retrieved through our Google News search. All retrieved articles were screened for relevance and to identify duplicates. The screening process did not consider quality or scope of coverage but was only performed to ensure that the retrieved articles (1) were not already in the corpus of articles, and (2) covered the original CNODES study (ie, were not false positives). Only English language articles were considered. This resulted in a dataset of 26 media articles and 3 publications produced by CNODES (the original CMAJ article, a press release, and a podcast produced by CMAJ of an interview with the study authors [Multimedia Appendix 1]). The texts of the articles were extracted and all 29 articles (26 media articles and 3 reference CNODES sources) were stored on a cloud-based server. All text preprocessing and analysis, as described below, were completed in Eclipse (Standard Luna-R), Microsoft Visual Studio 2013, and Python 3.7 (NLTK 3.2.1 and TextStat 0.3.1 libraries).

### Natural Language Processing

NLP is, generally, the ability of computers to analyze and manipulate natural language text or speech to provide an understanding of the text and answer questions about its contents. Different studies have demonstrated the application of NLP to information retrieval in a variety of areas such as question answering, social media text mining, and decision support systems [27-29]. Mendonça et al showed that encoding clinical data in patient documents using NLP techniques, along with clinical rules, can help identify health care–associated pneumonia in infants [30]. In a similar study, Dublin et al used only radiograph reports of previous cases with pneumonia to train their system to classify reports as consistent with pneumonia, inconsistent with pneumonia, or requiring manual review [31]. Knirsch et al utilized NLP methods to encode radiology reports which, along with other data in the patient repository, help detect patients who should be isolated but were not identified using the normal protocols [32].

In a different study, Wang et al combined text mining techniques with statistical analysis and patient electronic health records to detect adverse drug events. They applied NLP techniques to narrative discharge summaries to identify the safety of drugs throughout their entire lifecycle [33]. McTaggart et al adopted an NLP approach to analyze and transform large volumes of collected prescriptions (about 100 million per annum) into regular structured information on medication dose instructions [34].

These studies, and many more, show that NLP is an interdisciplinary area that includes a variety of computational techniques that, alone or in combination with other approaches, can perform a diverse set of tasks and applications. Along with the main purpose of this study, we leveraged various text mining techniques to analyze media articles (each technique explored in detail below):

1. Frequent words analysis to study the occurrence of words in each article and cluster, recognize the pattern of the most frequently used words, and investigate how the articles and clusters differ.
2. Term frequency-inverse document frequency (TF-IDF) weighting to calculate the closeness and/or separation between the articles through cosine similarity and Euclidean distance.
3. Hierarchical agglomerative clustering (HAC) to group (ie, cluster) similar articles together and to compare them with the original CNODES study.
4. Readability scales to calculate readability and analyze how easily the articles can be read and understood by an average reader.

### Data Cleaning and Text Preprocessing

NLP consists of 3 general steps: (1) text collection; (2) preprocessing; and (3) text analysis. Preprocessing is a crucial yet often undervalued part of the process and is key to the performance and accuracy of any text analysis [35,36]. Links, advertisements, and all multimedia components (eg, images, figures, and videos) that are not informative or related to the content of the article were removed [37].

The next step in preprocessing was the removal of stop words. Stop words (eg, conjunctions, prepositions, and articles) are uninformative, frequently occurring words that do not carry much meaning and do not contribute to the differentiation between documents [38]. We used simple automated text-searching techniques to remove any words of a standard English stop word list [39] (including 627 words) from all the collected media articles.

The final preprocessing step was to perform stemming. Stemming is the process of connecting different words that are derivatives of the same root (eg, *student*, *studies*, and *studied* are various forms of their stem, *study*) [40]. A stemming algorithm conflates all words with the same root to a common form. Stemming, compared with full word representations, improves the indexing time (ie, the time to create the dictionary and calculate the Vector Space Model (VSM) representation) in an information retrieval system by reducing the size of the dictionary (ie, index file) by 20%-50%. In addition, a shorter list of index terms helps to improve the relevancy of the retrieved documents [41-44].

There are different algorithms for stemming. In this study, we used Porter stemming [45], which is the most widely used stemming algorithm for different languages, including English. The Porter stemming algorithm is independent from the context and has significantly reduced the complexity of the rules associated with suffix removal [46]. It is worth mentioning that, to avoid any duplication, Porter stemming transforms all the words to lowercase and then calculates the stems.

### Frequent Words Analysis

The purpose of the frequent words analysis was to provide an overall summary of the content of the media articles and to compare the content of the different articles—and the clusters identified later in the analysis—to learn more about the texts and the areas of their focus. These findings will help to identify how and why the clusters are different and refine further analyses [47].

Although frequent words analysis can provide a valuable broad overview of the content of the documents, this approach does not provide much insight into the differences between documents, as common words tend to be common across all media outlets. To provide deeper insight into the relationships between media articles, we looked at how the articles might cluster together based on the content of their coverage.

### Article Clustering

The objective of article clustering was to identify patterns in coverage of the CNODES study. Using a 3-step process of TF-IDF weighting, similarity calculation, and HAC, we identified 3 potential clusters of similar media coverage and used the frequent words analysis to provide insight into how these clusters might have differed in their language and coverage choices.

### Term Frequency-Inverse Document Frequency Weighting

We used TF-IDF weighting in our analysis to gain insight into what makes individual articles unique. TF-IDF values represent the frequency of the words in a specific document relative to the frequency of that word over the entire corpus of documents [48,49]. The following equation depicts how the TF-IDF values are calculated in which *w_i,j_* is the weight for term *i* in document *j, N* is the number of documents in the corpus, *tf_i,j_* is the frequency of appearance of term *i* in document *j,* and *df_i_* is the frequency of term *i* in the corpus [50]:

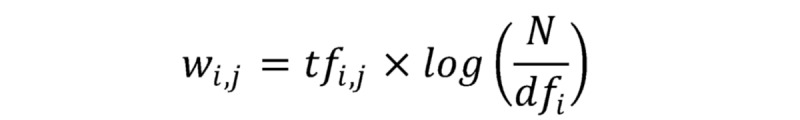



TF-IDF values were calculated for all unique terms (1-grams) and the combinations of 2 sequential terms (2-grams) from the corpus using the above weighting equation and stored in an *n x k* matrix—where each row represents an article (n=29) and each column (k=6158) represents a 1 or 2-gram. This is a standard VSM representation that prepares the data to calculate similarity between the documents.

Like most information retrieval systems, we considered multiword phrases (ie, 2-grams) as some phrases can be more meaningful and informative than individual terms. For example, in our study, the phrase *pregnancy prevention* can distinguish articles and find a degree of similarity between the collected documents better than 2 single terms *pregnancy* or *prevention*. In the calculations, we merged the combination of any 2 words in sequence (ie, 2 words that appear together) as a new phrase (ie, 2-grams) and included it in the VSM.

### Similarity Calculations

A similarity measure reflects the degree of closeness between 2 articles using a single numeric value [51]. We chose cosine similarity as it is easy to calculate and interpret and is commonly used in the NLP literature [52]. Cosine similarity returns a value between 0 and 1, where 2 documents with a similarity value of 1 are regarded as identical, and a value of 0 implies no similarity between the documents [51]. The result of the similarity calculations is a symmetric *n x n* similarity matrix (in our case, n=29).

### Hierarchical Agglomerative Clustering

In this study, we chose HAC to group the similarity matrix into groups of similar documents because of the flexibility of hierarchical approaches in the desired number of clusters, its efficiency for small datasets, and the feasibility of graphical representation of the results through a tree-like structure called a dendrogram [53,54].

In agglomerative clustering, cutting branches of the dendrogram at a selected height (cut-off point) defines the resulting clusters. Selecting the best cut-off point depends on a variety of parameters such as the desired number of clusters, the granularity of the categories, or the acceptable distance between the entities within the clusters [55,56].

We used Euclidean distance in the construction of the HAC clusters as it is more appropriate in this environment than the cosine similarity, but all the similarity values presented in this study are cosine similarity.

### Readability Analysis

The final objective of our analysis was to measure the readability [57] of the articles covering our initial study. Health literacy describes the extent to which one is able to acquire, interpret, and comprehend health information and services to make informed health decisions; the reading level of health information will either enable or impede its consumption [58,59]. Readability may be influenced by a variety of factors: the writing style, the clarity of words and sentences, and/or the degree to which a given text is compelling and comprehensible, based on a reader’s reading skill, prior knowledge, and motivation [60-63]. Although the average American reads at an 8th grade level, the American Medical Association and National Institutes of Health recommend that patient and health information be written at or below a 6th grade level [64-66].

There are a variety of ways to measure the readability of a text. Friedman and Hoffman-Goetz [67] found high concurrent validity and correlation between the various readability formulas, but no specific formula is accepted as the gold standard for assessing readability or reading ease of health information [68].

We used 9 well-formalized readability formulas (Table 1) to study the readability of the media articles. Multimedia Appendix 2 further elaborates the readability formulas and the scores. Readability measures were developed using TextStat 0.3.1 library (Bansal and Aggarwal, MIT) in Python Package Index 3.4.4.

**Table 1:** Readability formulas. C: number of characters; D: number of complex words; E: number of easy (not-complex words); P: number of polysyllables; S: number of sentences; W: number of words; Y: number of syllables; AC: average number of characters per 100 words; AS: average number of sentences per 100 words.

Readability score	Score type	Key statistical features	Formula
Flesch Reading Ease (FRES)	Numeric score (0-100)	Word length and sentence length	FRES=206.83 - 1.015 x (W/S) - 84.6 x (Y/W)
Flesch-Kincaid Grade (FKRA)	US grade level	Word length and sentence length	FKRA=0.39 x (W/S) – 11.8 x (Y/W) – 15.59
Gunning Fog Index (FOG)	US grade level	Number of complex words	FOG=0.4 x [ (W/S) + 100 x (D/W)]
Simple Measure of Gobbledygook Index	US grade level	Number of complex words	SMOG=1.0430 x √(P x 30/S) + 3.1291
Automated Readability Index (ARI)	US grade level	Number of characters	ARI=4.71 x (C/W) + 0.5 x (W/S) – 21.43
Coleman Liau Index (CLI)	US grade level^a^	Number of characters	CLI=0.0588 x AC + 0.296 x AS – 15.8
Linsear Write Index (LWI)	US grade level	Sentence length, number of polysyllables	(1) Find a 100-word sample from your writing; (2) Calculate Val=[E+(3×D)]/S; (3) If Val >20, then LWI=Val/2; (4) If Val ≤ 20, then LWI=(Val-2) / 2;
Dale-Chall Readability Score (DCRS)	Numeric score (0-9.9)	Number of difficult words	DCRS=0.1579 x (D/S) + 0.0496 x (W/S)
Text Standard	US grade level		A voting system among the other metrics: the reading level that is most prevalent (the mode) among the other metrics calculated.

^a^The terms in the table are stemmed versions of the actual terms (for example, us represents various forms of the verb use, and pregnanc stands for pregnancy).Grade level may also be understood as the number of years of formal education needed to understand a given text, particularly when the level exceeds the typical range of US grades (e.g. 1-12). For example, grades 13-16 suggest undergraduate training, 17-18 graduate training, and 19+ professional qualification.[63,67]

## Results

### Overview of Retrieved Articles

In total, 29 articles, including 26 media articles and 3 CNODES reference articles, comprised the corpus of documents for this study, and were represented in a VSM. The articles were of varying length: from 13 to 51 sentences, or 227 to 1011 words. The combined vocabulary of all articles contained 7745 unique terms (out of 11,263 total terms that appeared in the entire dataset). There was an average of 35 sentences, 740 words, and 1380 syllables per article, with an average of 30.9% (229/740) of the words being complex—words with 3 or more syllables that do not belong to a list of 3000 familiar words [69].

### Frequent Words Analysis

*Pregnanc* (stem of pregnancy) is the most frequent individual term among all the text with 344 occurrences, followed by *isotretinoin* and *studi* (stem of study, studies, etc) with 306 and 245 occurrences, respectively. *Preganc prevent* (stem of pregnancy prevention) and *birth defect* are the most recurrent 2-grams with the frequency of 74 and 63.

Table 2 shows British Columbia (BC), 1 of the 4 provinces that was included in the study, appeared 35 times in the entire corpus. However, the other study provinces (not shown in Table 2), Saskatchewan, Ontario, and Manitoba, appeared 43, 40, and 26 times, respectively.

Excluding those published by CNODES, only 2 articles (8% of the sources) mentioned or acknowledged CIHR, the study’s funder. *Health Canada* appeared in 13 and some variant of the phrase *conflict of interest* occurred in only 1 article beyond the CNODES articles.

**Table 2:** Top 10 most frequent vocabulary terms (1-grams and 2-grams).

1-grams	Frequency	Ratio	2-grams	Frequency	Ratio
pregnanc	344	0.031	pregnanc prevent	74	0.007
isotretinoin	306	0.027	birth defect	63	0.006
studi	245	0.022	birth control	48	0.004
drug	226	0.020	pregnanc test	40	0.004
women	188	0.017	women take	39	0.003
us	165	0.015	prevent program	37	0.003
birth	163	0.014	British Columbia	35	0.003
research	135	0.012	live birth	33	0.003
treatment	123	0.011	pregnanc rate	33	0.003
acn	118	0.010	isotretinoin user	31	0.003

### Similarity and Cluster Analysis

**Figure 2:**
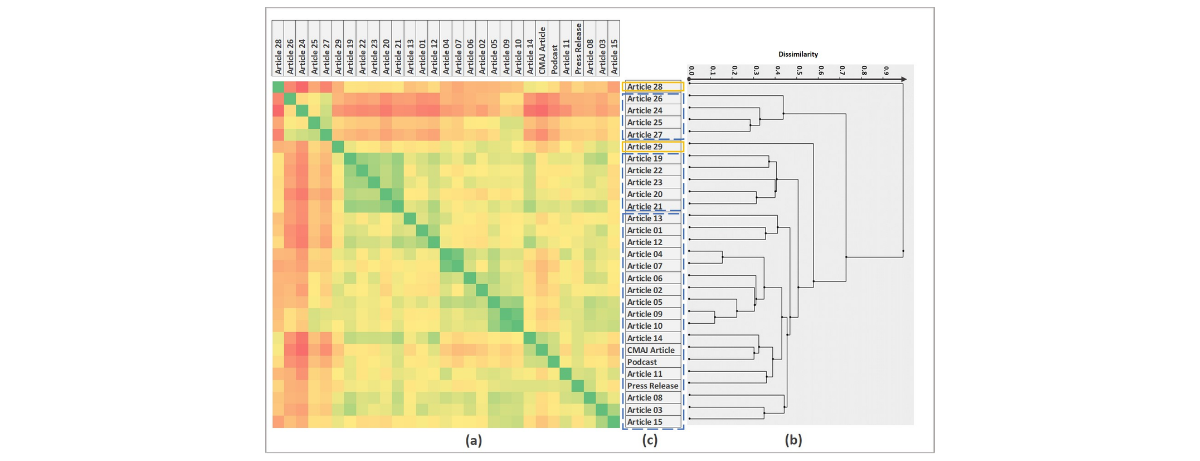
Cosine similarity values (between 0 and 1) between the media articles and CNODES publications, including CMAJ article, podcast, and press release article using TF-IDF calculations. Resulting dendrogram of hierarchical agglomerative clustering. Three clusters and 2 singletons, resulting from a cutoff point of 0.5. CMAJ: *Canadian Medical Association Journal*; CNODES: Canadian Network for Observational Drug Effect Studies; TF-IDF: term frequency-inverse document frequency.

The resulting values of cosine similarity calculations and HAC are presented in Figure 2. In the similarity matrix, green cells represent higher values of similarity (maximum of 1.0) between the articles; the similarity decreases as we move to the red side of the spectrum (minimum of 0.0). Using the similarity matrix and the dendrogram, we chose a cutoff in the dendrogram of 0.5, resulting in 3 distinct clusters. As the similarity values show, articles 28 and 29 are not significantly similar to any of the articles in the corpus. Consequently, they do not fit in any clusters. Articles 24 to 27 are similar to each other (with similarity values of 0.68 and above) but different from the remaining articles. Articles 19 to 23 are highly similar to each other, and articles 1 to 15 have higher values of similarity. These groups of articles were combined using HAC and form the 3 clusters: Cluster 1 (with 18 articles), Cluster 2 (with 5 articles), and Cluster 3 (with 4 articles).

Further examination of the nature of the articles in each cluster showed Cluster 1, in addition to the 3 CNODES publications, included national and international news websites such as Reuters, CBC, The Globe and Mail, National Post, and CTV. Cluster 1 also included health-specific websites such as Medical Daily, Medical News Today, MD Magazine, and Medscape Medical News. The articles composing Cluster 3 were from regional news websites including CBC British Columbia and The Globe and Mail British Columbia. Articles in Cluster 2 did not include traditional news media outlets, but rather health-related and general interest websites (Science Daily, Parent Herald, and Science 2.0).

**Figure 3:**
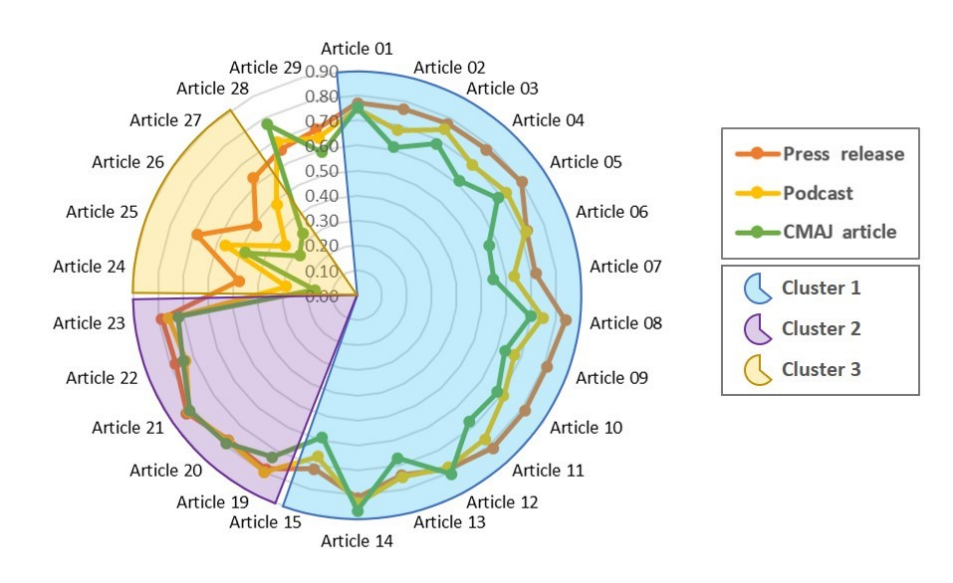
Trend of similarity (cosine similarity) between the media articles and the CNODES publications: CMAJ article, podcast, and press release.

Figure 2 also shows that the 3 CNODES publications—the CMAJ article, podcast, and press release—are highly similar to each other, with similarity values of 0.81 and above. Because of that degree of resemblance, the media articles maintain the same trend of similarity to the CNODES publications—an article which is similar to any of the 3 CNODES publications is similar to the other 2 and vice versa. Figure 3 depicts this steady trend of similarity by comparing the similarity of each media article to the CNODES publications separately.

### Frequent Words Analysis Within the Clusters

In addition to studying the nature of the websites that published the media articles, we found that analysis of the frequent words within the clusters provides insight into how and to what extent the clusters are different. Table 3 shows the 5 most common terms within each cluster, along with specific clinical terms that we selected *a priori* to measure across the clusters.

*Pregnancy* and *isotretinoin* are the most common terms in the articles of Clusters 1 and 2, while these 2 terms are not among the top 10 frequent terms of Cluster 3. In addition, Clusters 1 and 2 have 6 frequent terms in common, while only 2 frequent terms of Cluster 3 (*studi* and *drug*) appear in the top 10 frequent terms of Clusters 2 and 3. Frequent words analysis within the clusters, in accordance with the similarity matrix (see Figure 2), implies Clusters 1 and 2 are more similar to each other than to Cluster 3.

Table 3 reveals the articles in Clusters 1 and 2 preferred to use *isotretinoin* (ranked 2) rather than Accutan (ranked 32 and 20, respectively), which is a brand name of *isotretinoin*; *isotretinoin* and *Accutan* were the 54th and 12th most frequent words, respectively, among the articles in Cluster 3. These rankings show the articles in Cluster 3 have chosen to focus on the brand name of the drug, rather than its generic name.

Table 3 shows Clusters 1 and 2 have focused on *patient* and *treatment*, while these concepts are not in a high position in the articles of Cluster 3. *Birth defect* has a relatively constant focus across the clusters. Cluster 3 did not discuss *fetal*, *fetal risk*, *fetal abnormality*, or *miscarriage* at all. *Acne* is in a significantly lower position for Cluster 3 (ranked 60th).

There is an overlap between the clinically important terms and the most frequent terms for each cluster. Hence, the top 5 most frequent terms of each cluster include the phrases that are not already mentioned in the 5 clinically most important terms. For example, since the 1st, 2nd, 3rd, and 7th most frequent terms of Cluster 1 are among the top 5 clinically important terms, the top 5 most frequent terms of Cluster 1 include the next 5 most frequent terms (the 4th, 5th, 6th, 8th, and 9th frequent terms of the cluster).

**Table 3:** Most common terms, both overall and within each cluster.

Cluster^a^			Cluster 1		Cluster 2		Cluster 3		Singleton 28		Singleton 29
**5 clinically most important terms**											
	Isotretinoin	240 (2)^b^		49 (2)		6 (48)		7 (2)		4 (7)	
	Accutan^c^	46 (32)		12 (20)		17 (11)		—^d^		3 (12)	
	pregnanc	263 (1)		57 (1)		8 (33)		12 (1)		5 (3)	
	drug	166 (3)		28 (6)		23 (7)		1 (50)		8 (1)	
	birth	127 (7)		22 (9)		8 (33)		1 (50)		5 (3)	
**Top 5 most frequent terms^a^** **of cluster 1**											
	studi	160 (4)		31 (3)		42 (2)		6 (3)		6 (2)	
	Us	142 (5)		18 (12)		3 (107)		—		2 (17)	
	women	139 (6)		30 (4)		14 (14)		—		5 (3)	
	treatment	101 (8)		16 (14)		3 (107)		2 (16)		1 (39)	
	patient	94 (9)		11 (24)		9 (27)		2 (16)		1 (39)	
**Top 5 most frequent terms^a^** **of cluster 2**											
	studi	160 (4)		31 (3)		42 (2)		6 (3)		6 (2)	
	women	139 (6)		30 (4)		14 (14)		—		5 (3)	
	prevent	72 (16)		30 (4)		4 (74)		5 (4)		2 (17)	
	canadian	62 (22)		27 (7)		6 (48)		2 (16)		1 (39)	
	take	55 (28)		24 (8)		9 (27)		—		3 (12)	
**Top 5 most frequent terms^a^** **of cluster 3**											
	research	82 (13)		9 (27)		43 (1)		—		1 (39)	
	studi	160 (4)		31 (3)		42 (2)		6 (3)		6 (2)	
	health	66 (19)		1 (411)		38 (3)		—		—	
	Data	35 (44)		—		38 (3)		—		—	
	said	56 (27)		7 (41)		33 (5)		—		4 (7)	

^a^The terms in the table are stemmed versions of the actual terms (for example, us represents various forms of the verb use, and pregnanc stands for pregnancy).

^b^Top 5 most frequent terms of each cluster exclude the 5 clinically important terms.

^c^The first number in the cells shows the frequency of occurrence of the term, and the second number in the parenthesis shows the ranking of the terms among all the termt in that cluster.

^d^Empty cells (represented with a —) are the terms that do not appear in the respective cluster/singleton.

### Readability Analysis

Overall, 9 readability formulas were calculated for each article in the corpus. Different readability formulas consider different variables in the calculations and measure readability from distinct perspectives (see Table 1).

**Figure 4:**
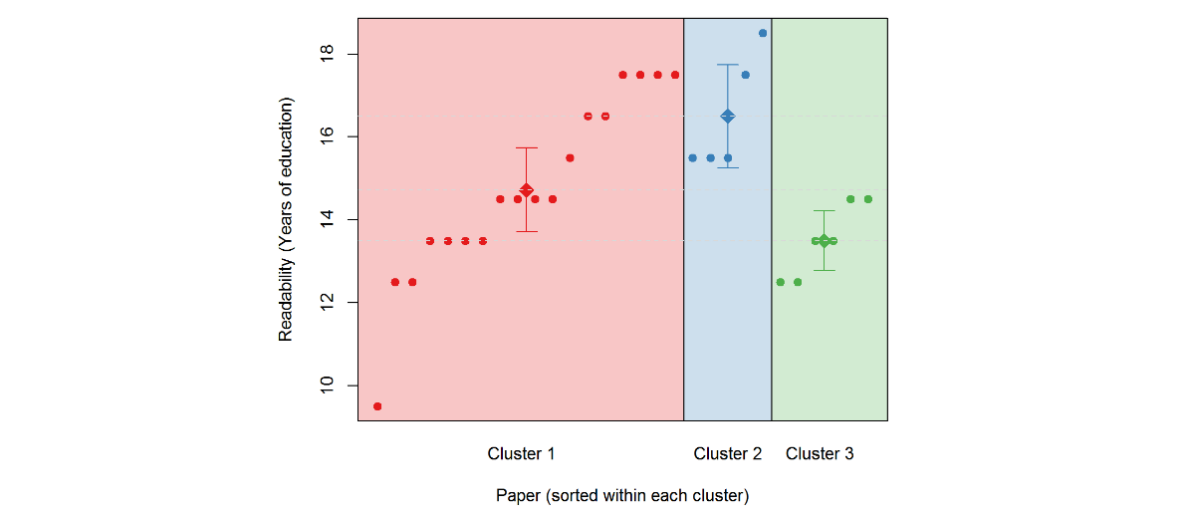
Distribution of readability levels of articles based on text-standard measure.

All calculated readability scores are above United States grade 10. Text standard scores, which represent the most prevalent reading level among all the formulas, ranged between 12 and 18, except for one article with a readability level of 9. Figure 4 demonstrates the distribution of readability levels of articles based on text-standard measure.

Table 4 shows reading ease based on calculating the average of each readability score for the articles within the clusters. On average, the articles in Cluster 3 were the easiest to read, followed by the articles in Clusters 1 and 2.

**Table 4:** Average readability level of each cluster.

Cluster.	Flesch Reading Ease	Flesch-Kincaid Grade	Gunning Fog Index	SMOG Index	Automated Readability Index	Coleman Liau Index	Linsear Write Index	Dale-Chall Readability Score	Text Standard
Cluster 1	40.78	13.02	15.19	15.58	15.21	14.47	13.27	9.87	16th grade
Cluster 2	29.89	14.74	16.59	16.92	16.76	15.97	10.62	10.67	17th grade
Cluster 3	49.19	11.35	13.75	14.33	13.35	13.32	8.85	9.39	14th grade
Singleton 28	36.79	12.50	12.99	15.90	15.20	16.82	13.75	8.82	12th grade
Singleton 29	49.55	11.70	15.11	15.00	15.40	14.74	8.08	9.87	14th grade

## Discussion

### Overall Results

Our NLP analysis of media coverage showed that the interpretation of the CNODES isotretinoin study [16] was diverse, with significant variations in content, language, areas of focus, and reading level. The primary focus of the media coverage was pregnancy and pregnancy prevention, but this focus was not consistent across all articles. Some articles focused more on the disease, drug, and treatment, while others emphasized the study and the related government regulations.

Regardless of the method used to calculate reading level, the overall reading levels were too high for the average North American reader, where the target reading level should be grades 6-8 [64-66]. Consequently, these media stories may have failed to reach many potential isotretinoin users of child-bearing potential. Even when the reading level calculations were re-run under different scenarios, such as reducing the complexity of complex words (eg, isotretinoin) through substitutions with shorter terms (eg, drug), the reading levels remained well above recommended reading levels.

Our results were similar to other studies which documented high reading levels for plain language communications of scientific advice. For example, in a study of 53 qualified health claims on food and dietary supplement labels, which are regulated by the United States Food and Drug Administration, the Flesch-Kincaid grade level ranged from 5.37 to 30.30, with 77% above a grade 9 reading level [70].

Overall disclosure of funders was low, with only 2 media articles naming CIHR as the funding organization. Financial disclosure is especially important in journalism covering pharmaceuticals where various conflicts of interest may exist [71,72].

The CNODES study was covered by the Canadian newspaper, The Globe and Mail, which averages 3.1 million print and digital readers on a typical weekday. It received coverage from both national television (CBC and CTV) and more specialized media with niche audiences such as iPolitics, which covers federal, provincial, and international politics and policies. The study also received international coverage from Thomson Reuters (www.thomsonreuters.com), which covers a broad range of topics in media markets around the world. The articles varied in length, ranging from approximately 200 to 1000 words, in large part due to standard word limits set by each media outlet [73].

### Words Used

We had expected a significant overlap between some of the articles, with the potential for articles to be reprinted in different venues; overall, the words used in each media report were less similar than expected. Although there were commonalities between the articles, there was little evidence of republication or wholesale duplication of articles. We were not able to easily discern if certain articles were informed by others. Although the original CNODES source material did seem to influence the content of each article, each article author (or set of authors) clearly applied their own spin to the content. It is possible that if there had been more media coverage, patterns of duplication might have emerged. However, we have no evidence to suggest there were any patterns of reprinting in this corpus.

The clusters varied in the extent of overlap with our original press release and the top words used. Documents 28 and 29 had less similarity to the other articles in the corpus. It is interesting that document 28 was the American Pharmacist, which would likely employ science writers, and document 29 was the CBC in Saskatchewan, where they had direct access to one of the authors of the study who resided in Saskatchewan and was able to provide additional information from the Saskatchewan perspective. It has been noted that several publications reprint the press releases they receive without additional comment or contextualization and many media outlets are vertically integrated, although these integrations were not reflected in our analysis. It is interesting that the 10th most frequent 2-gram was *isotretinoin user*, which is an epidemiologic term and comes directly from the research study with specific definitions. Of note, health care providers (eg, physicians, pharmacists, and nurses) did not come up in the top 10 words. Instead, the focus seemed to be on women using isotretinoin, many of whom were not also dispensed birth control pills, and the protective actions they should be taking rather than on what health care providers or policymakers should be doing.

Table 3 shows the words by cluster. It is interesting that acn (stem of acne) came up in the top 10 only in Cluster 1. In media articles, it is useful to set the context: isotretinoin is approved only for severe cystic acne, although it is frequently used off-label. The top words in Cluster 2 were isotretinoin and pregnancy, so perhaps they were focusing more on the effects of isotretinoin than the purpose of it. Cluster 3 had research and study as their top 2 results, reflecting that they are focusing on reporting the results of the study conducted, rather than trying to consume and translate the results themselves. Clusters 1 and 2 used isotretinoin more frequently than Accutane (a common brand name for the isotretinoin product), while Cluster 3 used Accutane more frequently, reflecting different approaches on how to communicate the drug to the audience.

Omission of specific parts of the media release were surprising, such as the lack of disclosure around study funding (CIHR) and potential conflicts of interest. Although many of the articles did mention Health Canada, better reporting about the study team would have provided better context for the research and information on potential competing interests.

Table 4 shows the variability of reading levels, both between equations and across clusters. Regardless of which readability measure was used, each cluster showed a readability level that was too high, making it difficult for some patients to comprehend the material. The National Institutes of Health and American Medical Association suggest that health education material be written at a 6th to 8th grade level [64,74]. Readability calculations like these are not the only approach to measuring health literacy and are known to have shortcomings [10,67,75]. We looked at readability but not a reader’s motivation to read one of the media documents or their ability to comprehend it [67]. We also did not examine numeracy, which is critical in the drug safety literature. In future, we will broaden our investigations of other aspects of health literacy, combining readability with the ability to find, process, and understand information, and to integrate these concepts with other sources of information to support health decision making [3,76]. Finally, although we looked at digital media coverage and examined specific aspects of health literacy, we did not examine electronic health literacy, which is an important concept.

### Limitations

Our study takes a novel approach to tracking the media coverage of academic research after it has been published and is an important part of growing the knowledge translation component of the CNODES project, but it has its shortcomings. Our search, although comprehensive from a keyword perspective, was limited to media outlets that published on the internet. We did not search the websites of individual newspapers, with the assumption that our general Google News search would capture all relevant mentions. We did not evaluate pictures that were associated with the media articles, the way in which numbers were reported, or links to other resources. We did not consider the expertise of the journalists, specifically, whether there was a difference in the reporting between health journalists and general assignment reporters. We did not examine the length of the media article beyond its influences on reading level, so there may be further insights to be gleaned from comparing article length with specific aspects such as funding source and article positioning (eg, front page). Finally, although we believe we have captured all meaningful media coverage of our study, our data capture window was relatively short, we did not use a commercial news aggregator, and we did not specifically examine gray literature, so there is always the potential that we have missed some media articles.

We are currently not able to speak to who the articles may have deemed responsible for the original study results (ie, poor pregnancy prevention guideline adherence) or to determine the quality of the media report [8]. This type of insight is nuanced and difficult to achieve using NLP techniques, but should be explored more in future work as these insights would be valuable. We have also not analyzed the way in which the media stories were received, understood, and used by patients, health care providers, and policymakers, nor what additional information these individuals may have used to support their decision making [3].

There are many known limitations to using reading-level metrics [10,67,75]; thus, it is possible we are overestimating how difficult it may be to read the media coverage. It is important to understand that reading level is only one way to evaluate readability, and only one aspect of many to consider when communicating health information effectively [77].

Placement within the media content is an important determinant of consumption and could provide an indication of an article’s perceived value. In a digital age, these factors can change significantly over time and between users. We were unable to process this information. We did not specifically examine if independent sources (such as other researchers) were used by journalists to inform context and study validity, or whether patients, voluntary health organizations, or drug regulatory agencies provided their perspectives. We were unable to identify if the journalist was an employee of the news organization or if the article came from a news wire service or syndicated service. We also did not examine if a link to the original CMAJ article was provided.

We did not consider the quality of the coverage in terms of source. Although we subjectively evaluated the coverage to deem it as relevant or not, an objective measure of quality (such as the DISCERN tool [78]) or popularity could both assess the quality of the coverage and provide another document-level metric to understand the full extent of media coverage. Future work in this area should consider these factors.

### Recommendations and Implications for Practice

It is important for researchers to understand how their research is presented by the media. Our analysis demonstrates that there is little consistency in how this is done using a peer-reviewed research article, even when accompanied by a crafted press release and outreach by the primary authors. If there are potentially controversial or sensitive issues arising from the research that need to be presented carefully, then the narrative around these issues should be appropriately constructed in the wording of the press releases and an effort needs to be made to monitor how the information is being translated in real time as it is disseminated. The reading levels of the media covering research can be quite high; more efforts should be made to simplify the press releases and other knowledge translation materials generated from the research so that journalists can more easily present the research in an accessible manner. Researchers can assist journalists by identifying other aspects of their research such as broader context and limitations [13].

### Future Study

Improving the reading levels of CNODES’ dissemination efforts, particularly outside of academic literature, could improve the ability of CNODES to reach key target audiences (eg, health care providers, decision makers). Further work is needed to develop automated media coverage analysis so that researchers can quickly and efficiently identify how their research is being covered and what is and is not being consumed, with the potential to react to it in real time and correct any potential misinterpretations by media outlets. Future research will need to augment readability approaches with other approaches, such as the use of mental model research [79], to inform communications strategies. Expanding on the analysis with sentiment and qualitative analyses would also be valuable as there are insights into sentiment and attribution that were not explored in this paper. The approach to document similarity we took in this paper considered the documents as a whole, but there is potential for articles to overlap in content from certain sections of the document, while adding their own local or audience-specific context to a common theme. Future research into topic modeling [80] could help identify themes that are common across documents, to contrast with document-specific themes.

Although this study focused solely on the content of the words presented in the articles, future research should incorporate the use of photos, captions, hyperlinks, and multimedia to form a more complete picture of how a study was presented. Due to the changing and various ways of presenting information on the Web, this kind of project would require careful and deliberate planning and would be difficult to do on a retrospective basis.

Extending this study to social media coverage would be a valuable addition; there are large and meaningful discussion sections accompanying some of the articles in this study (eg, doc09). Our research group has studied the altmetrics of our research on social media [81]. Combining these two research arms in a single stream could provide more nuanced results.

### Conclusions

This study has demonstrated that NLP can be a valuable tool in understanding how research is conveyed to the public through digital media. Through NLP, we identified significant variations in the coverage of our research and what parts of our publications journalists focused on. We demonstrated how readability calculations can be applied to media coverage. Our future work will look at expanding our methods to better understand how our research is consumed by the media.

## Appendix

Multimedia Appendix 1 List of articles (26 media articles, 3 Canadian Network for Observational Drug Effect Studies reference publications).

Multimedia Appendix 2 Readability scales.

## Abbreviations

BC: British Columbia
CIHR: Canadian Institutes of Health Research
CMAJ: Canadian Medical Association Journal
CNODES: Canadian Network for Observational Drug Effect Studies
HAC: hierarchical agglomerative clustering
NLP: natural language processing
TF-IDF: term frequency-inverse document frequency
VSM: vector space model

## References

[1] Storino A, Castillo-Angeles M, Watkins AA, Vargas C, Mancias JD, Bullock A, Demirjian A, Moser AJ, Kent TS (2016). Assessing the accuracy and readability of online health information for patients with pancreatic cancer. JAMA Surg.

[2] Fox S, Duggan M (2013). Pew Research Center.

[3] Hanson H, O'Brien N, Whybrow P, Isaacs JD, Rapley T (2017). Drug breakthrough offers hope to arthritis sufferers: qualitative analysis of medical research in UK newspapers. Health Expect.

[4] (2012). Statistics Canada.

[5] Walsh-Childers K, Braddock J, Rabaza C, Schwitzer G (2018). One step forward, one step back: changes in news coverage of medical interventions. Health Commun.

[6] CAJ Ethics Advisory Committee (2011). The Canadian Association of Journalists.

[7] (2018). National NewsMedia Council.

[8] Zeraatkar D, Obeda M, Ginsberg JS, Hirsh J (2017). The development and validation of an instrument to measure the quality of health research reports in the lay media. BMC Public Health.

[9] (2019). The National Academies of Sciences, Engineering, Medicine.

[10] (2007). Food and Drug Administration.

[11] Schwartz LM, Woloshin S (2003). On the prevention and treatment of exaggeration. J Gen Intern Med.

[12] Bubela TM, Caulfield TA (2004). Do the print media 'hype' genetic research? A comparison of newspaper stories and peer-reviewed research papers. Can Med Assoc J.

[13] Dentzer S (2009). Communicating medical news--pitfalls of health care journalism. N Engl J Med.

[14] Moynihan R, Bero L, Ross-Degnan D, Henry D, Lee K, Watkins J, Mah C, Soumerai SB (2000). Coverage by the news media of the benefits and risks of medications. N Engl J Med.

[15] Haneef R, Yavchitz A, Ravaud P, Baron G, Oransky I, Schwitzer G, Boutron I (2017). Interpretation of health news items reported with or without spin: protocol for a prospective meta-analysis of 16 randomised controlled trials. BMJ Open.

[16] Henry D, Dormuth C, Winquist B, Carney G, Bugden S, Teare G, Lévesque LE, Bérard A, Paterson JM, Platt RW, CNODES (Canadian Network for Observational Drug Effect Studies) Investigators (2016). Occurrence of pregnancy and pregnancy outcomes during isotretinoin therapy. Can Med Assoc J.

[17] Suissa S, Henry D, Caetano P, Dormuth CR, Ernst P, Hemmelgarn B, Lelorier J, Levy A, Martens PJ, Paterson JM, Platt RW, Sketris I, Teare G, Canadian Network for Observational Drug Effect Studies (CNODES) (2012). CNODES: the Canadian Network for Observational Drug Effect Studies. Open Med.

[18] (2012). Canadian Institutes of Health Research.

[19] Lammer EJ, Chen DT, Hoar RM, Agnish ND, Benke PJ, Braun JT, Curry CJ, Fernhoff PM, Grix AW, Lott IT (1985). Retinoic acid embryopathy. N Engl J Med.

[20] Rosa FW (1983). Teratogenicity of isotretinoin. Lancet.

[21] Crijns HJ, Straus SM, Gispen-de Wied C, de Jong-van den Berg LT (2011). Compliance with pregnancy prevention programmes of isotretinoin in Europe: a systematic review. Br J Dermatol.

[22] Azoulay L, Oraichi D, Bérard A (2006). Patterns and utilization of isotretinoin for acne from 1984 to 2003: is there need for concern?. Eur J Clin Pharmacol.

[23] Honein MA, Moore CA, Erickson JD (2004). Can we ensure the safe use of known human teratogens? Introduction of generic isotretinoin in the US as an example. Drug Saf.

[24] Shin J, Cheetham TC, Wong L, Niu F, Kass E, Yoshinaga MA, Sorel M, McCombs JS, Sidney S (2011). The impact of the iPLEDGE program on isotretinoin fetal exposure in an integrated health care system. J Am Acad Dermatol.

[25] Choi JS, Koren G, Nulman I (2013). Pregnancy and isotretinoin therapy. Can Med Assoc J.

[26] (2019). CMAJ Article Metrics.

[27] Chowdhury GG (2005). Natural language processing. Ann Rev Info Sci Tech.

[28] Mohammadhassanzadeh H, Shahriari HR (2013). Prediction of user's trustworthiness in web-based social networks via text mining. ISC Int J Inf Security.

[29] Demner-Fushman D, Chapman WW, McDonald CJ (2009). What can natural language processing do for clinical decision support?. J Biomed Inform.

[30] Mendonça EA, Haas J, Shagina L, Larson E, Friedman C (2005). Extracting information on pneumonia in infants using natural language processing of radiology reports. J Biomed Inform.

[31] Dublin S, Baldwin E, Walker RL, Christensen LM, Haug PJ, Jackson ML, Nelson JC, Ferraro J, Carrell D, Chapman WW (2013). Natural Language Processing to identify pneumonia from radiology reports. Pharmacoepidemiol Drug Saf.

[32] Knirsch CA, Jain NL, Pablos-Mendez A, Friedman C, Hripcsak G (1998). Respiratory isolation of tuberculosis patients using clinical guidelines and an automated clinical decision support system. Infect Control Hosp Epidemiol.

[33] Wang X, Hripcsak G, Markatou M, Friedman C (2009). Active computerized pharmacovigilance using natural language processing, statistics, and electronic health records: a feasibility study. J Am Med Inform Assoc.

[34] McTaggart S, Nangle C, Caldwell J, Alvarez-Madrazo S, Colhoun H, Bennie M (2018). Use of text-mining methods to improve efficiency in the calculation of drug exposure to support pharmacoepidemiology studies. Int J Epidemiol.

[35] Ahonen H, Heinonen O, Klemettinen M, Verkamo AI (1997). CiteSeerX.

[36] Uysal AK, Gunal S (2014). The impact of preprocessing on text classification. Inf Process Manag.

[37] Mierswa I, Wurst M, Klinkenberg R, Scholz M, Euler T (2006). YALE: Rapid Prototyping for Complex Data Mining Tasks. Proceedings of the 12th ACM SIGKDD international conference on Knowledge discovery and data mining.

[38] Yao Z, Ze-wen C (2011). Research on the Construction and Filter Method of Stop-word List in Text Preprocessing. 2011 Proceedings of the Fourth International Conference on Intelligent Computation Technology and Automation.

[39] (2017). Git Hub.

[40] Ali NH, Ibrahim NS (2012). Porter stemming algorithm for semantic checking.

[41] Lovins JB (1968). Development of a stemming algorithm. Mech Translat Comp Linguistics.

[42] Hajeer SI, Ismail RM, Badr NL, Tolba MF, Hassanien AE, Fouad MM, Manaf AA, Zamani M, Ahmad R (2017). A new stemming algorithm for efficient information retrieval systems and web search engines. Multimedia Forensics and Security: Foundations, Innovations, and Applications.

[43] Baeza-Yates R, Ribeiro-Neto B (1999). Modern Information Retrieval.

[44] Sembok T, Ata BM, Bakar ZA (2011). A rule and template based stemming algorithm for Arabic language. Int J Math Mod Meth Appl Sci.

[45] Porter MF (1980). An algorithm for suffix stripping. Program.

[46] Willett P (2006). The Porter stemming algorithm: then and now. Program.

[47] Danneman N, Heimann R (2014). Social Media Mining With R.

[48] Aizawa A (2003). An information-theoretic perspective of tf–idf measures. Inf Process Manag.

[49] Ramos J (2003). Using tf-idf to determine word relevance in document queries. Proceedings of the First Instructional Conference on Machine Learning.

[50] Zhang W, Yoshida T, Tang X (2011). A comparative study of TF*IDF, LSI and multi-words for text classification. Expert Syst Appl.

[51] Huang A (2008). Similarity measures for text document clustering. Proceedings of the Sixth New Zealand Computer Science Research Student Conference.

[52] Dhillon IS, Modha DS (2001). Concept decompositions for large sparse text data using clustering. Mach Learn.

[53] Deshpande R, Vaze K, Rathod S, Jarhad T (2014). Comparative study of document similarity algorithms and clustering algorithms for sentiment analysis. Int J Emerg Trends Technol Comput Sci.

[54] Zepeda-Mendoza ML, Resendis-Antonio O, Dubitzky W, Wolkenhauer O, Yokota H, Cho KH (2013). Hierarchical agglomerative clustering. Encyclopedia of Systems Biology.

[55] Janssens F, Zhang L, Moor BD, Glänzel W (2009). Hybrid clustering for validation and improvement of subject-classification schemes. Inf Process Manag.

[56] Langfelder P, Zhang B, Horvath S (2008). Defining clusters from a hierarchical cluster tree: the Dynamic Tree Cut package for R. Bioinformatics.

[57] DuBay WH (2004). Impact Information.

[58] Nielsen-Bohlman L, Panzer AM, Kindig DA, Institute of Medicine (US) Committee on Health Literacy (2004). Health Literacy: A Prescription To End Confusion.

[59] Health Gov.

[60] Bailin A, Grafstein A (2001). The linguistic assumptions underlying readability formulae: a critique. Lang Commun.

[61] Klare GR (1963). The Measurement of Readability.

[62] Hargis G (2000). Readability and computer documentation. ACM J Comput Doc.

[63] McLaughlin GH (1969). SMOG grading - a new readability formula. J Reading.

[64] Weiss BD (2003). National Center for Farmworker Health: On-Line Library.

[65] (2017). MedlinePlus.

[66] Eltorai AE, Han A, Truntzer J, Daniels AH (2014). Readability of patient education materials on the American Orthopaedic Society for Sports Medicine website. Phys Sportsmed.

[67] Friedman DB, Hoffman-Goetz L (2006). A systematic review of readability and comprehension instruments used for print and web-based cancer information. Health Educ Behav.

[68] Smith T (2016). The problems with current readability methods and formulas: missing that usability design. Proceedings of the 2016 IEEE International Professional Communication Conference.

[69] (2017). Character Count.

[70] Berhaupt-Glickstein A, Hallman WK (2017). Communicating scientific evidence in qualified health claims. Crit Rev Food Sci Nutr.

[71] Stassen W (2016). Semantic Scholar.

[72] Moynihan R, Heath I, Henry D (2002). Selling sickness: the pharmaceutical industry and disease mongering. Br Med J.

[73] (2009). Kaiser Family Foundation.

[74] Hansberry DR, Agarwal N, Gonzales SF, Baker SR (2014). Are we effectively informing patients? A quantitative analysis of on-line patient education resources from the American Society of Neuroradiology. AJNR Am J Neuroradiol.

[75] Eloy JA, Li S, Kasabwala K, Agarwal N, Hansberry DR, Baredes S, Setzen M (2012). Readability assessment of patient education materials on major otolaryngology association websites. Otolaryngol Head Neck Surg.

[76] Nutbeam D, Boxall A (2008). What influences the transfer of research into health policy and practice? Observations from England and Australia. Public Health.

[77] Sykes S, Wills J, Rowlands G, Popple K (2013). Understanding critical health literacy: a concept analysis. BMC Public Health.

[78] (1997). DISCERN.

[79] Bruine de Bruin W, Bostrom A (2013). Assessing what to address in science communication. Proc Natl Acad Sci USA.

[80] Blei DM (2012). Probabilistic topic models. Commun ACM.

[81] Gamble JM, Traynor RL, Gruzd A, Mai P, Dormuth CR, Sketris IS (2018). Measuring the impact of pharmacoepidemiologic research using altmetrics: a case study of a CNODES drug-safety article. Pharmacoepidemiol Drug Saf.

