# Correction: Using Natural Language Processing to Examine the Uptake, Content, and Readability of Media Coverage of a Pan-Canadian Drug Safety Research Project: Cross-Sectional Observational Study

**DOI:** 10.2196/20211

**Published:** 2020-06-23

**Authors:** Hossein Mohammadhassanzadeh, Ingrid Sketris, Robyn Traynor, Susan Alexander, Brandace Winquist, Samuel Alan Stewart

**Affiliations:** 1 Dalhousie University Halifax, NS Canada; 2 Nova Scotia Health Authority Halifax, NS Canada; 3 University of Saskatchewan Saskatoon, SK Canada

In “Using Natural Language Processing to Examine the Uptake, Content, and Readability of Media Coverage of a Pan-Canadian Drug Safety Research Project: Cross-Sectional Observational Study” (JMIR Form Res 2020;4(1):e13296), several errors were noticed.

Susan Alexander’s degree was incorrectly noted in the original article; it has been revised from *MSc* to *MHI*.

Figures 3 and 4 were incorrectly positioned in the original article. Figure 3 was displaying in the position of Figure 4 and Figure 4 was displaying in the position of Figure 3. This has now been corrected in the manuscript. Figure 3 is the circle graph, depicting the similarity of all articles to the three original articles; Figure 4 is the distribution chart, depicting the distribution of readability levels of articles (y-axis) based on text-standard measures.

The figure legends for Figures 3 and 4 were also incorrect in the original article.

Figure 3's legend has been revised from:

Steady trend of similarity (cosine similarity) between the media articles and the CNODES publications: CMAJ article, podcast, and press release

to:

Trend of similarity (cosine similarity) between the media articles and the CNODES publications: CMAJ article, podcast, and press release

Figure 4's legend has been revised from:

Similarity of all articles to three original articles (press release, podcast, and CMAJ article)

to:

Distribution of readability levels of articles based on text-standard measure

Additionally, due to a technical error, there was a discrepancy between the HTML and PDF versions of the original article. In the HTML version, the inline equation incorrectly displayed as a figure and was assigned a figure number, causing the figures to be numbered incorrectly, and the placement of the circle graph was also incorrect. This has now been corrected.

Finally, there were errors in Multimedia Appendix 1 and Multimedia Appendix 2 in the original article.

In Multimedia Appendix 1, the title has been revised from:

Appendix 2. List of articles (26 media articles, 3 CNODES reference publications)

to:

Multimedia Appendix 1. List of articles (26 media articles, 3 CNODES reference publications)

In Multimedia Appendix 2, the title has been revised from:

Appendix 1. Readability scales

to:

Multimedia Appendix 2: Readability scales

The correction will appear in the online version of the paper on the JMIR website on June 23, 2020, together with the publication of this correction notice. Because this was made after submission to PubMed, PubMed Central, and other full-text repositories, the corrected article has also been resubmitted to those repositories.

## Appendix

Multimedia Appendix 1 List of articles (26 media articles, 3 CNODES reference publications).

Multimedia Appendix 2 Readability scales.

