# Supplementary material for: Using Natural Language Processing to Examine the Uptake, Content, and Readability of Media Coverage of a Pan-Canadian Drug Safety Research Project: Cross-Sectional Observational Study
Source: JMIR Form Res. 2020 Jan 14;4(1):e13296. doi: 10.2196/13296 (PMC6996767; doi:10.2196/13296)
Supplement: Multimedia Appendix 1 [file formative_v4i1e13296_app1.docx]

**Multimedia Appendix 1**. List of articles (26 media articles, 3 CNODES reference publications)

| **Cluster** | **Document Number** | **Headline** | **Media Outlet / Publisher** | **Article URL** |
| --- | --- | --- | --- | --- |
| 1 | doc01 | Women using popular acne drug aren't following advice to avoid pregnancy | Reuters | <http://www.reuters.com/article/us-health-acne-pregnancy-idUSKCN0XM28O> |
| 1 | doc02 | Acne drug Accutane's harm to fetus a worry despite prevention efforts | CBC | <http://www.cbc.ca/news/health/acne-accutane-pregnancy-1.3551549> |
| 1 | doc03 | Canadian women uneducated on birth defect risk of acne drug Accutane, study finds | The Globe and Mail/health | <http://www.theglobeandmail.com/life/health-and-fitness/health/canadian-women-uneducated-on-birth-defect-risk-of-acne-drug-accutane-study-finds/article29749016/> |
| 1 | doc04 | Using a common acne drug improperly? You could end up with pregnancy complications, miscarriage: study | Global News | <http://globalnews.ca/news/2660565/using-a-common-acne-drug-improperly-you-could-end-up-with-pregnancy-complications-miscarriage-study/> |
| 1 | doc05 | Canadian women still getting pregnant while on Accutane, despite risks of birth defects: study | National Post | <http://news.nationalpost.com/news/0426-na-accutane> |
| 1 | doc06 | Accutane users not following contraceptive rules despite birth defect risks: study | CTV | <http://www.ctvnews.ca/health/accutane-users-not-following-contraceptive-rules-despite-birth-defect-risks-study-1.2873987> |
| 1 | doc07 | Using a common acne drug improperly? You could end up with pregnancy complications, miscarriage: study | Gossip Monthly | <http://gossip-monthly.com/health/using-a-common-acne-drug-improperly-you-could-end-up-with-pregnancy-complications-miscarriage-study/43379> |
| 1 | doc08 | Acne drug taken during pregnancy leading to miscarriages, defects: Study | iPolitics | <http://ipolitics.ca/2016/04/25/acne-drug-taken-during-pregnancy-leading-to-miscarriages-defects-study/> |
| 1 | doc09 | Government-delayed study on drug safety costs hundreds of lives | rabble.ca | <http://rabble.ca/babble/canadian-politics/government-delayed-study-on-drug-safety-costs-hundreds-lives> |
| 1 | doc10 | Canadian women still getting pregnant while on Accutane, despite risks of birth defects: study | Ottawa Citizen | <http://www.ottawacitizen.com/health/canadian+women+still+getting+pregnant+while+accutane+despite/11876211/story.html> |
| 1 | doc11 | Acne Treatment With Accutane Causes Birth Defects, Yet Many Patients Still Don’t Follow Contraceptive Rules | Medical Daily | <http://www.medicaldaily.com/acne-treatment-accutane-birth-defects-contraception-ipledge-383308> |
| 1 | doc12 | Pregnancy guidelines for harmful acne drug not adhered to by many women | Medical News Today | <http://www.medicalnewstoday.com/articles/309516.php> |
| 1 | doc13 | Women Ignore Contraception Rules While on Accutane | MD Magazine | <http://www.hcplive.com/medical-news/women-ignore-contraception-rules-while-on-accutane> |
| 1 | doc14 | Pregnancy Prevention Program in Acne Drug Users Ineffective | Medscape Medical News | <http://www.medscape.com/viewarticle/862430> |
| 1 | doc15 | Women in Canada Using Accutane Acne Treatment Uneducated About Its Risk for Birth Defect, Study Finds | Science World Report | <http://www.scienceworldreport.com/articles/38878/20160428/women-in-canada-using-accutane-acne-treatment-uneducated-about-its-risk-for-birth-defect-study-finds.htm> |
| 1 | doc16 | Occurrence of pregnancy and pregnancy outcomes during isotretinoin therapy | CMAJ | <http://www.cmaj.ca/content/early/2016/04/25/cmaj.151243> |
| 1 | doc17 | Occurrence of pregnancy and pregnancy outcomes during isotretinoin therapy (PODCAST) | CMAJ | <http://www.cmaj.ca/content/early/2016/04/25/cmaj.151243/suppl/DC2> |
| 1 | doc18 | Poor contraceptive practices are leading to pregnancies in users of isotretinoin, a drug that causes birth defects | CNODES | - |
| 2 | doc19 | Contraception Safety Program for Acne Drug Failing in Canada | Health Day | <http://consumer.healthday.com/kids-health-information-23/acne-news-3/safety-program-for-acne-drug-failing-in-canada-study-finds-710221.html> |
| 2 | doc20 | Accutane: Despite Birth Defects Risk, 50 Percent Of Canadian Women Don't Comply With Requirements | Science 2.0 | <http://www.science20.com/news_articles/accutane_despite_birth_defects_risk_50_percent_of_canadian_women_dont_comply_with_requirements-171231> |
| 2 | doc21 | Birth defects, pregnancy terminations, miscarriages in users of acne drug | Science Daily | <https://www.sciencedaily.com/releases/2016/04/160425141528.htm> |
| 2 | doc22 | Pregnancy Prevention: Canadian Women Ignoring Guidelines In Using Harmful Acne Drug | Parent Herald | <http://www.parentherald.com/articles/39387/20160426/pregnancy-prevention-canadian-women-ignoring-guidelines-using-harmful-acne-drug.htm> |
| 2 | doc23 | Accutane contraception prevention program failing in Canada | Digital Journal | <http://www.digitaljournal.com/life/health/accutane-contraception-prevention-program-failing-in-canada/article/463879> |
| 3 | doc24 | Mass firing delayed study on birth defects caused by Accutane | The Globe and Mail | <http://www.theglobeandmail.com/news/british-columbia/mass-firing-delayed-study-on-birth-defects-caused-by-accutane/article29756717/> |
| 3 | doc25 | Privacy probe stalled research on acne drug's dangers: NDP | Times Colonist | <http://www.timescolonist.com/news/local/privacy-probe-stalled-research-on-acne-drug-s-dangers-ndp-1.2239242> |
| 3 | doc26 | B.C. health ministry firings caused delay in Accutane-pregnancy study | CBC British Columbia | <http://www.cbc.ca/news/canada/british-columbia/b-c-health-firings-accutane-pregnancy-1.3553813> |
| 3 | doc27 | Privacy breach in B.C. health ministry led to freeze on medical research data | The Globe and Mail-BC | <http://www.theglobeandmail.com/news/british-columbia/privacy-breach-in-bc-health-ministry-led-to-freeze-on-medical-research-data/article29767108/> |
| Singleton | doc28 | Occurrence of pregnancy and pregnancy outcomes during isotretinoin therapy | American Pharmacist Association | <http://www.pharmacist.com/occurrence-pregnancy-and-pregnancy-outcomes-during-isotretinoin-therapy> |
| Singleton | doc29 | Acne drug Accutane causing problems for Saskatchewan pregnancies | CBC Saskatchewan | <http://www.cbc.ca/news/canada/saskatchewan/acne-drug-saskatchewan-pregnancy-1.3552317> |
