# Supplementary material for: Using Natural Language Processing to Examine the Uptake, Content, and Readability of Media Coverage of a Pan-Canadian Drug Safety Research Project: Cross-Sectional Observational Study
Source: JMIR Form Res. 2020 Jan 14;4(1):e13296. doi: 10.2196/13296 (PMC6996767; doi:10.2196/13296)
Supplement: Multimedia Appendix 2 [file formative_v4i1e13296_app2.docx]

**Multimedia Appendix 2.** Readability scales

| **Scale** | **Interpretation of scores** | **Citation/URL** |
| --- | --- | --- |
| Flesch Reading Ease (1948) | - Higher scores indicate material that is easier to read (e.g. 90-100, Very Easy) - Lower numbers indicate material that is difficult to read (e.g. 30-49, Difficult) | <http://www.readabilityformulas.com/flesch-reading-ease-readability-formula.php> |
| Flesch-Kincaid Grade Level Score (1976) | - A score of 5.0 indicates 5^th^ grade-school level of readability; a score of 9.3 indicates a 9^th^-grade level of readability. | <http://www.readabilityformulas.com/flesch-grade-level-readability-formula.php> |
| Gunning’s Fog Index (1952) | - The ideal score for readability is 7 or 8; anything above 12 is too difficult for the general public. | <http://www.readabilityformulas.com/gunning-fog-readability-formula.php> |
| SMOG Index (1969) | - Based on the number of polysyllables found in a reading material: polysyllables 1 – 6 = Grade 5; 7 – 12 = Grade 6; 13 – 20 = Grade 7; 21 – 30 = Grade 8; 31 – 42 = Grade 9; 43 – 56 = Grade 10; 57 – 72 = Grade 11; 73 – 90 = Grade 12 ; 91 – 110 = Grade 13 ; 111 – 132 = Grade 14; 133 – 156 = Grade 15; 157 – 182 = Grade 16; 183– 210 = Grade 17; 211 – 240 = Grade 18. | <http://www.readabilityformulas.com/smog-readability-formula.php> |
| Automated Readability Index (ARI) (1967) | - 5-6 years old: Kindergarten - 6-7 years old: Grade 1 - 17-18 years old: Grade 12 - 18-22 years old: College | <http://www.readabilityformulas.com/automated-readability-index.php> |
| Coleman Liau  Index (1975) | - A grade level of 10.6 is roughly appropriate for a 10 – 11^th^ grade high school student. | <http://www.readabilityformulas.com/coleman-liau-readability-formula.php> |
| Linsear Write Index (1977) | - Calculates if a 100-word sample is at 9^th^ or 10^th^ grade level. | <http://www.readabilityformulas.com/linsear-write-readability-formula.php> |
| Dale-Chall  Readability Score (2017) | - <= 4.9: Grade 4 and Below - 5.0 to 5.9: Grades 5 - 6 - 6.0 to 6.9: Grades 7 - 8 | <http://www.readabilityformulas.com/new-dale-chall-readability-formula.php> |
| Text Standard | - US grade level, an average of the above grade reading levels | - |
